# Combinatorial drug screening on 3D Ewing sarcoma spheroids using droplet-based microfluidics

**DOI:** 10.1016/j.isci.2023.106651

**Published:** 2023-04-12

**Authors:** Romain Fevre, Gaëtan Mary, Nadia Vertti-Quintero, Aude Durand, Raphaël F.-X. Tomasi, Elaine Del Nery, Charles N. Baroud

**Affiliations:** 1 Laboratoire d’ Hydrodynamique (LadHyX), CNRS, EcolePolytechnique, InstitutPolytechnique de Paris, 91128 Palaiseau, France; 2 Institut Pasteur, Université Paris Cité, Physical microfluidics and Bioengineering, 25-28 Rue du Dr. Roux, 75015 Paris, France; 3 Okomera, iPEPS, the HealthTech Hub, Paris Brain Institute, HôpitalPitiéSalpêtrière, 75013 Paris, France; 4 Biophenics High-Content Screening Laboratory, Translational Research Department, PICT-IBiSA, Institut Curie, PSL Research University, 75005 Paris, France

**Keywords:** Drug delivery system, Molecular biology experimental approach, Cancer, Biological science instrumentation

## Abstract

Culturing and screening cells in microfluidics, particularly in three-dimensional formats, has the potential to impact diverse areas from fundamental biology to cancer precision medicine. Here, we use a platform based on anchored droplets for drug screening. The response of spheroids of Ewing sarcoma (EwS) A673 cells to simultaneous or sequential combinations of etoposide and cisplatin was evaluated. This was done by culturing spheroids of EwS cells inside 500 nL droplets then merging them with secondary droplets containing fluorescent-barcoded drugs at different concentrations. Differences in EwS spheroid growth and viability were measured by microscopy. After drug exposure such measurements enabled estimation of their IC50 values, which were in agreement with values obtained in standard multiwell plates. Then, synergistic drug combination was evaluated. Sequential combination treatment of EwS with etoposide applied 24 h before cisplatin resulted in amplified synergistic effect. As such, droplet-based microfluidics offers the modularity required for evaluation of drug combinations.

## Highlights

• A microfluidic protocol to subject cancer spheroids to drug combinations is developed
• Image analysis yields data on hundreds of individual spheroids per experimental run
• The device is benchmarked against standard plates and yields the same values of IC50
• Drug synergy was quantified for simultaneous and sequential exposure protocols

## Graphical abstract

**Figure:**
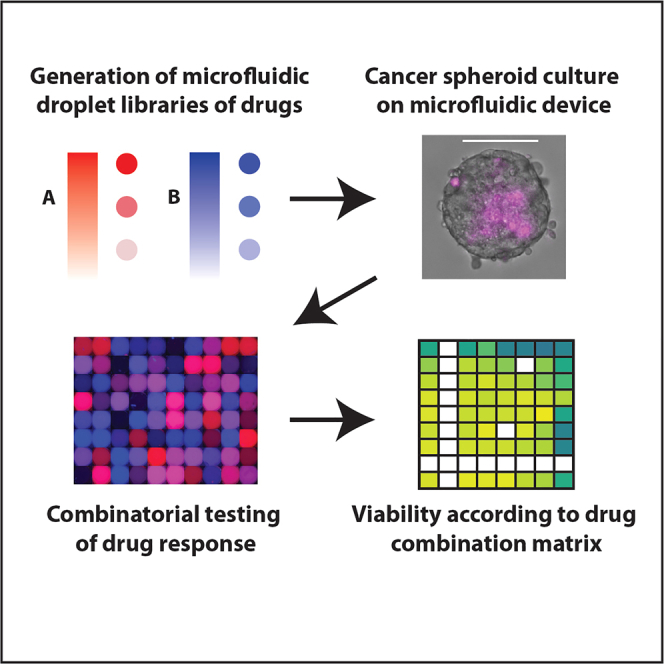

## Introduction

3D cell culture models have attracted considerable attention in the field of cancer research, particularly concerning their potential to increase the predictability of *in vivo* drug responses (reviewed in ref. ^1^^,^^2^). Tumor cell cultures grown as aggregates and spheroids demonstrate higher drug resistance to chemotherapeutics in comparison with 2D tumor cell cultures grown as monolayers,^3^^,^^4^^,^^5^^,^^6^ then revealing the crucial influence of cellular spatial organization and gene expression profiles on overall drug responses.^7^ Over the past few years, a plethora of methods and techniques for 3D cell cultures have been developed, including magnetic levitation,^8^ hanging drop-based methods,^9^^,^^10^^,^^11^^,^^12^ round bottom non-adherent microwells^13^ or droplet microfluidics.^14^

The successful adaptation of such 3D culture approaches for anti-cancer drug testing has become a powerful tool to better depict responses to currently used chemotherapies,^15^ novel immunotherapies,^16^^,^^17^ and in drug resistance studies.^3^ Although high-throughput screening (HTS) of single-agent therapeutics has also been successfully implemented in 96- and 384-well formats,^18^ it is not always feasible to adapt such platforms to study drug combinations, even for a reduced subset of anti-cancer drugs. Among the different 3D cultures methods, microfluidics is a promising one,^19^^,^^20^ because it can provide dynamical screens with drug cocktails and signaling molecules, where the concentration, timing, and duration of the fluidic delivery can be precisely controlled in an automated fashion.

Within the broad area of microfluidics, droplet-based systems have recently been developed for testing drug effects on individual cells or multicellular agregates, as recently reviewed in ref. ^14^. The droplet format allows a large number of independent experiments to be performed in parallel, by taking advantage of the encapsulation of the cells within isolated drops. On the other hand, droplets also introduce limitations on the duration of cell culture because of their limited volumes. Nevertheless, anchored droplets^21^ have been shown to allow multiplexed tests within a compact and easy to use device, both for chemical^22^ and cellular^23^ therapy models. In addition to this, the good integration of such microfluidic devices with microscopy techniques provides a method to obtain a large amount of data from a limited number of cells.

Here, we adapt a droplet-based microfluidic pipeline^22^ to allow drug combination studies using the Ewing sarcoma A673 cell line model.^24^ Chemotherapy remains indeed a fundamental treatment for patients with cancer, and particularly for Ewing sarcoma pediatric and adolescent patients.^25^ Drug resistance is a notorious factor that thwarts the effectiveness of current chemotherapeutic agents alone, including those currently used in the first-line chemotherapy. For this reason, drug combinations of such therapies are an important option to overcome resistance to single drug treatments and improve overall survival.^26^ Continued efforts are needed to implement cost-effective platforms allowing the evaluation of drug combinations as novel therapeutic strategies for these patients.

### Methodology

The purpose of this study is to implement a droplet-based microfluidic system to screen pairwise drug combinations on EwS spheroids within an array of droplets. Etoposide and cisplatin chemotherapies^27^ were employed to assess the response of EwS cells to drug treatments. These two drugs are well known chemotherapy drugs used as front-line cytotoxic therapy to treat several types of cancers, including pediatric cancers. They are both used in combination therapies to overcome drug-resistance and reduce toxicity. Etoposide is an anti-tumor drug that targets DNA topoisomerase II activities, thus leading to the production of DNA breaks and eliciting a response that affects several aspects of cell metabolisms^28^^,^^29^; cisplatin is a platinum-based alkylating agent able to disrupt DNA repair mechanisms, causing DNA damage, and subsequently inducing tumor cell death.^30^

**Figure 1:**
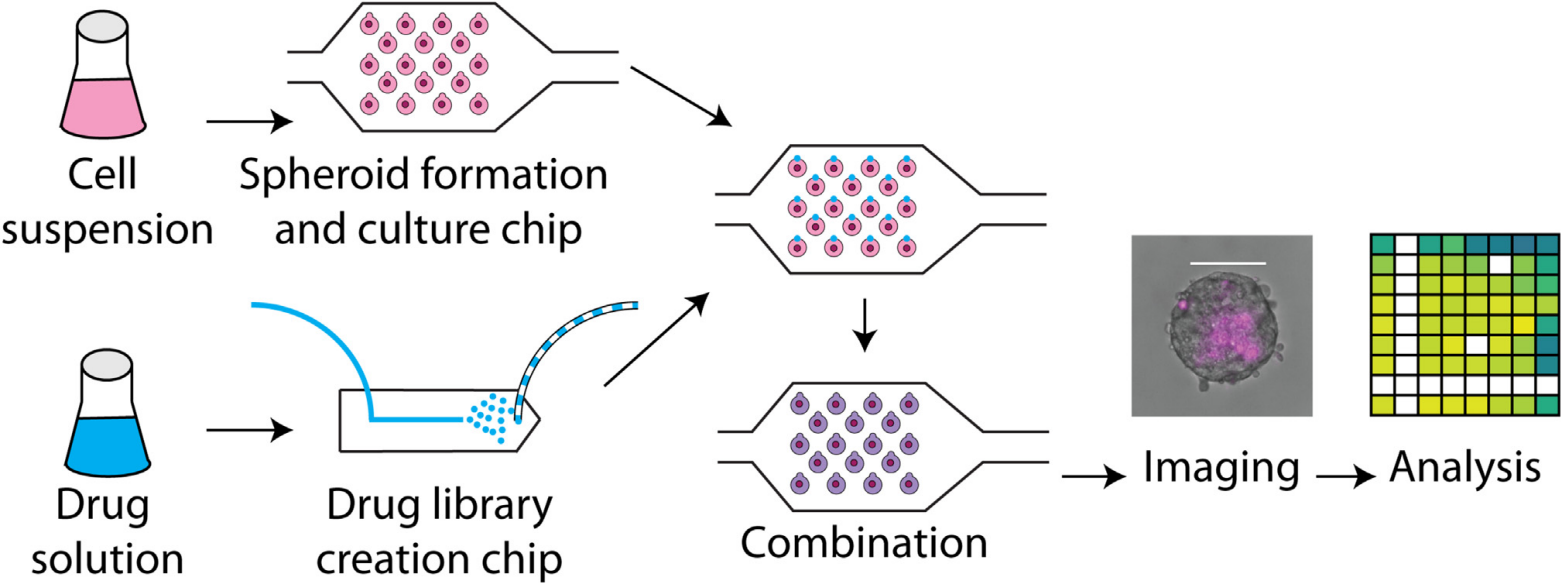
Experimental protocol for spheroid formation, culture and exposure to drugs EwS cells were suspended in culture medium and introduced to the culture microfluidic chip, where 3D spheroids were formed and cultured. In parallel, droplet drug libraries were created in a different microfluidic chip. Such drug droplets were introduced as secondary droplets to the culture chip, where they were fused to primary droplets containing spheroids, challenging in this manner the spheroids with the drugs. Subsequent systematic imaging and image analysis resulted in relevant time-dependent viability information. Scale bar represents 100 μm.

The microfluidic setup we used consisted of two different devices: a first device for the controlled formation and culture of EwS spheroids and a second one for the creation of a droplet drug library. Resulting droplets from both devices (containing either EwS spheroids or the drug library) were combined 1-to-1 inside one single device, where EwS spheroids’ viability was measured at later time points. The experimental workflow is depicted in Figure 1.

For the EwS spheroid formation step, GFP-expressing A673 cells were encapsulated into 500 nL droplets in the first microfluidic device, hereafter called ”culture chip”. Such droplets were formed and then captured in capillary anchors, in a similar manner as previously presented.^20^^,^^22^^,^^31^^,^^32^

In parallel to the spheroid formation and culture, solutions of drugs at different concentrations were encapsulated into 20 nL droplets in the second microfluidic device, hereafter called ”library chip”.^33^ Such droplets were collected *off-chip*, in a centrifuge tube, until they were needed. At a later time point, the library of cisplatin or etoposide-containing droplets was introduced into the culture chip containing the EwS spheroids. As a result, the drug droplets were captured by secondary anchors adjacent to the trapped spheroid-containing droplets. Then the droplet pairs were fused by means of chemically induced interface destabilization, to bring the two droplet contents in contact.^22^

Several imaging steps allowed information to be retrieved from each experiment. First the identity of individual spheroids was maintained over the course of the experiment because of their physical location on the microfluidic device. In parallel, knowledge of the applied drug concentration was retrieved from the combined droplets by a strategy of barcoding.^22^ Finally, the spheroids’ viability was measured over time, by including propidium iodide (PI) in each spheroid-containing droplet, to mark dead cells in each measurement time point. Images of EwS spheroids were automatically processed and analyzed to generate viability response curves as a function of drug concentrations. By obtaining images at different time points, relevant information on the efficacy of drug concentration and its dynamics was obtained. More detailed information on these steps is given in the following sections.

## Results

### Microfluidic platform for spheroid formation and culture

**Figure 2:**
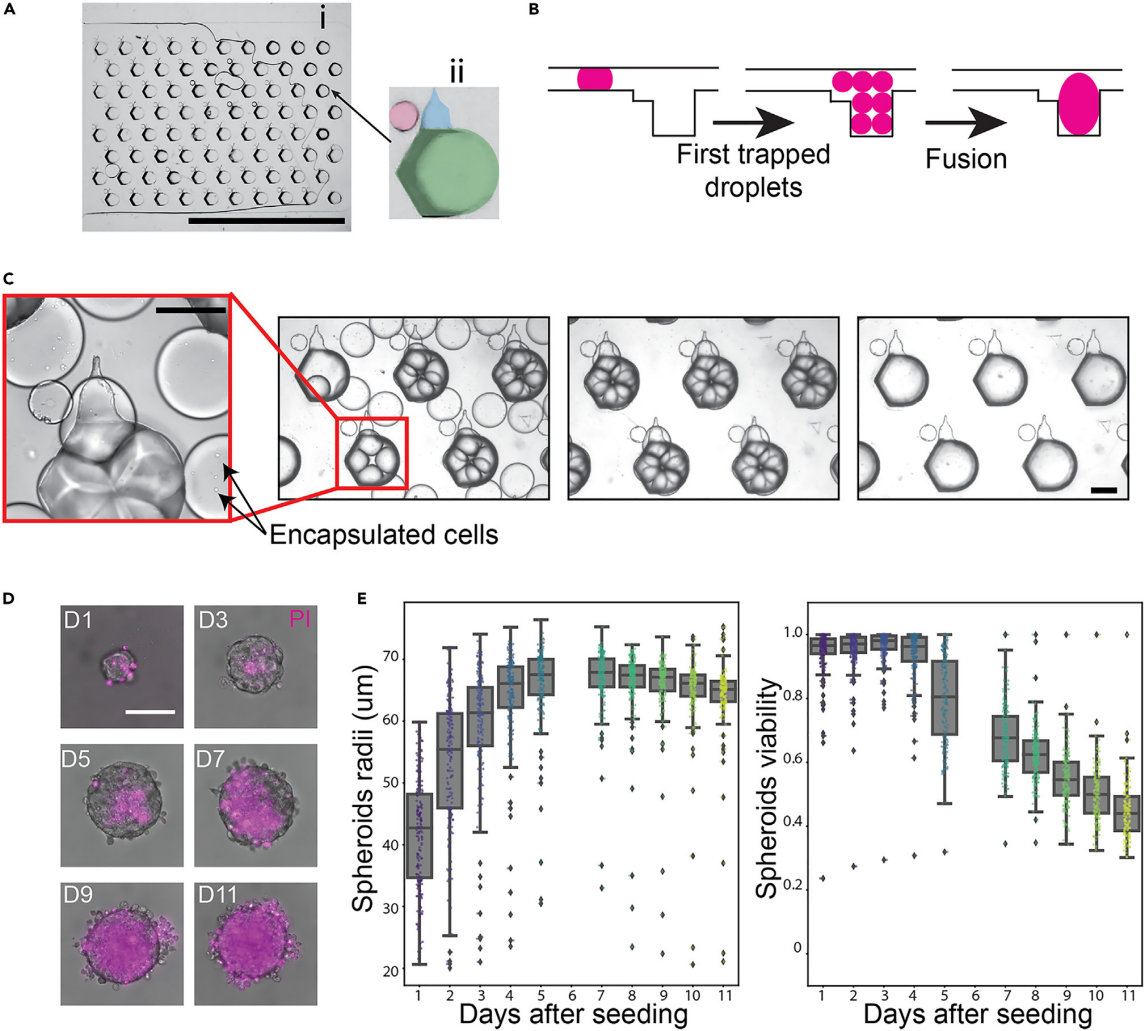
Microfluidic platform for spheroid formation and culture (A) Photography of (i) the array of traps for spheroid culture in the microfluidic chip, where (ii) each first trap (green) is adjacent to a secondary trap (blue) for a secondary droplet and a small pillar (red). Scale bar represents 1 cm. (B) Schematic of the steps toward spheroid formation and culture in the microfluidic traps: first droplets are generated and then trapped in first microfluidic traps. Then, we induce their fusion, which results in a larger droplet that fills up the microfluidic trap. (C) Picture of droplets containing cells in suspension following the steps shown in (B). Droplets are trapped in the microfluidic chamber; once the microfluidic chamber is filled, interface disruption is induced, forcing cells to fuse into the large drop. Scale bars represent 400 μm. (D) Time lapse of a spheroid, showing its growth and cell death (stained with PI dye) over days. Scale bar for all images is 100 μm. (E) Spheroids radii over time (in days) when culturing in droplets, as well as their viability over days.

The first step toward combinatorial drug screening on 3D EwS spheroids was the formation and culture of such spheroids. We adapted the microfluidic device previously presented by Tomasi et al.^22^ and Saint-Sardos et al.^32^: The culture microfluidic chip (Figure 2A) features a flow focusing injector for on-chip droplet formation and a large chamber for droplet trapping (Figure 2A (i)) where spheroids are formed and cultured. This culture zone is constituted by a 2D array of 80 anchors (Figure 2A (ii)). As previous designs, the device operates by modulation of droplet confinement, which is achieved by varying the channel depths of the microfluidic device to create the anchors.^21^ The primary part of the anchor ((ii), green) has an 800 μm diameter and depth (in addition to the 160 μm chamber height), which results in a very strong capillary trapping force applied on confined droplets. Meanwhile, the secondary part of the anchor ((ii), blue) has a 230 μm width and an 80 μm depth, providing a smaller capillary trapping force.^22^ The red circle in (ii) highlights a chamber pillar (250 μm width, 80 μm height) that strengthens the immobilization of the secondary droplets on the secondary part of the anchor. The spheroids were made by suspending the cells (volumetric concentration of $4.10^{5}$ cells/ml) in supplemented DMEM (10% of FBS, +1% of P/S), which were then introduced into the culture chip and dispersed into 45 nL droplets in a fluorinated oil phase (FC40 + 2% RAN). Each of these droplets contained around 20 cells and they were guided by the oil flow toward the culture zone of the chip. Primary traps, with total volume of about 540 nL, were big enough for trapping about 10 droplets.

Droplets were generated in the device until the full capacity of the primary traps was achieved, *i.e.* no more droplets could be trapped. Subsequently, the interface of the droplets was destabilized by introducing a solution of perfluorooctanol (PFO) in oil (FC40 + 20% PFO). After his oil exchange, the smaller droplets fused into a single larger droplet when bathing in the PFO solution for 1 to 3 min (Figures 2B and 2C). Subsequently, pure FC40 oil (without PFO) was flowed inside the microfluidic chip to remove any remaining emulsion destabilizer or untrapped droplets. This loading protocol resulted in around 200 cells per 540 nL droplet and the chip was then placed inside a cell incubator at $37^{\circ }$ C, 5% ${\mathrm{CO}}_{2}$ in between experiments. During this incubation time the cells in suspension inside the trapped droplets settled on the bottom interface where they aggregated to form a single spheroid per droplet.

The viability of cells within the spheroids was evaluated by adding PI (1 μM) into the primary droplet. The protocol consisted of imaging the spheroids once every 24 h and using image analysis to assess the viability using both the PI and GFP signals, as explained in detail in the STAR Methods section.

Before testing the drugs, the compatibility of the droplet-based culture with the spheroids was verified by measuring the growth and the viability of the spheroids in droplets over several days.^14^ The size and viability of 160 independent spheroids is shown in Figure 2E for a period of 11 days. The data indicate that the spheroids grow in size and their mean viability stays over 80% for the first 5 days, with the emergence of a necrotic core in the spheroids, as observed in Figure 2D. However a clear decrease in viability is measured after the seventh day of culture. In contrast with the previous measurements, the viability of spheroids grown in a smaller droplet volume (45 nL) is very low even at day 1, as shown in Figure S1. As a result the smaller droplets are not used in this study.

Analogous measurements for spheroids were performed in standard 96-well plates (shown in Figure S2 n=80). The chips in the microfluidic chips displayed slower growth and an increased mortality after day 5. Therefore the microfluidic experiments were considered to be physiologically relevant only in the first 5 days of culture.

### Droplet drug library production for dose-dependent toxicity screening

**Figure 3:**
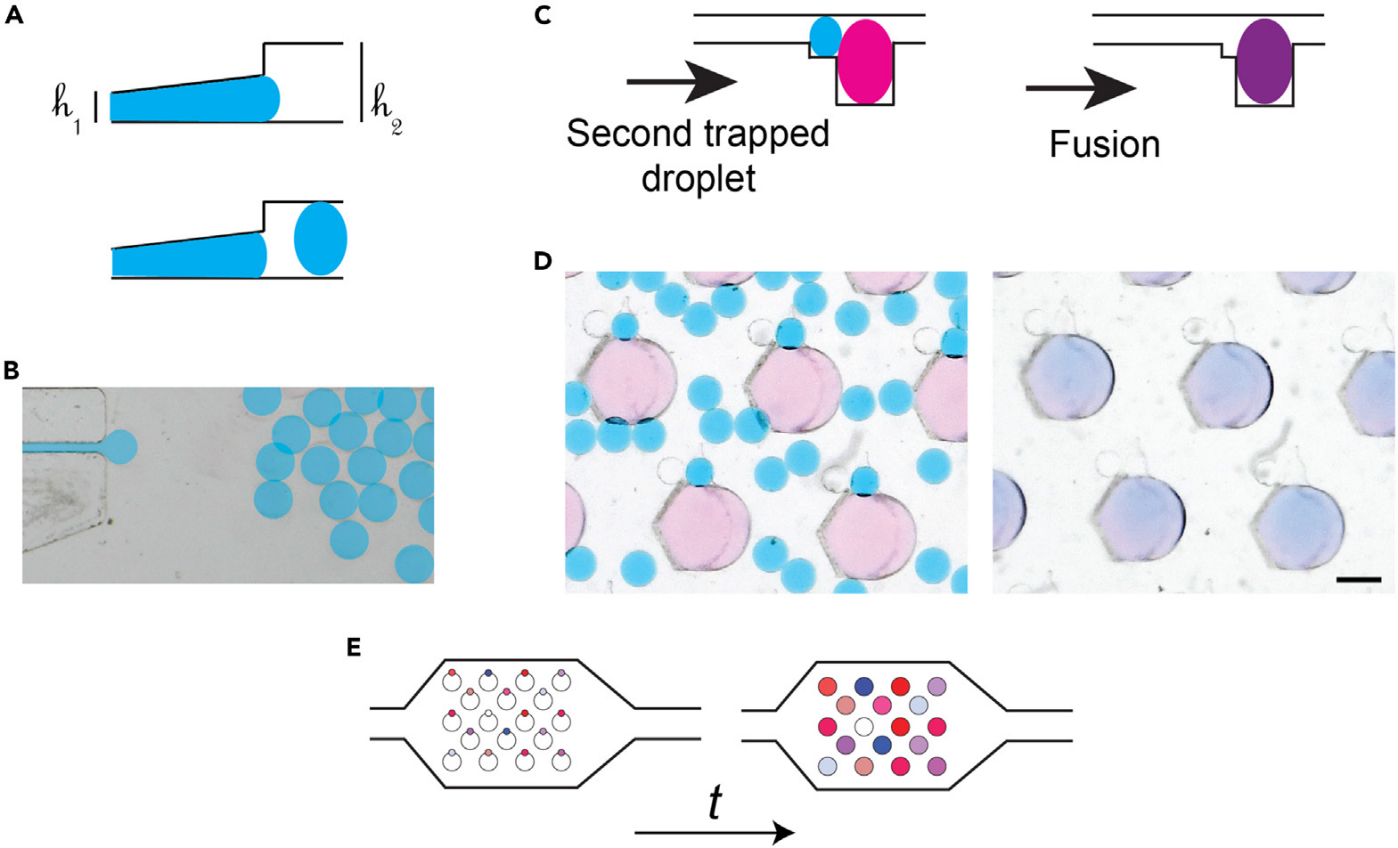
Droplet drug library production for toxicity screening (A) Schematic side view of the microfluidic channel geometry that allows for the droplet drug library production. (B) Top view of the chip used for creating secondary droplets. (C) Schematic side view of the steps toward droplet fusion: secondary droplets with a content of interest are trapped in secondary traps next to the primary trapped droplets, subsequently fusion on droplets is induced. (D) Photographs of droplets doing the steps shown in (c). Scale bar represents 400 μm. (E) Schematic of the consecutive steps toward addition of multiple secondary droplets to primary droplets.

A droplet drug library was produced by generating droplets with known drug concentrations in the library chip. This device features a sloping roof to apply a gradient of confinement to the immiscible interfaces, as shown in Figures 3A and 3B. These confinement gradients lead to the formation of a monodisperse emulsion of droplets with a high level of robustness and independently of the physical properties of the fluids.^33^^,^^34^ The aqueous droplets that were thus produced had a volume of 20 nL and were extracted into an external tube for storage off-chip.

In the library generation experiments the continuous oil phase was made of a fluorinated oil (HFE-7500 + 3% fluorosurfactant) and the dispersed aqueous phase was made of dilutions of either etoposide^28^^,^^29^ or cisplatin.^30^ Stock solutions of both drugs were prepared following manufacturer instructions: etoposide diluted in DMSO and cisplatine in water with 0.9% NaCl. Then, small volumes of dilutions (25 μL per concentration) at known concentrations were introduced into the microfluidic device for production of the droplet drug library. This library contained between 8,750 and 12,500 droplets each, representing between 7 and 10 concentrations of the drugs.

The droplet drug library was introduced in a random manner in the culture chip, where primary spheroid-containing droplets were already anchored (Figure 3C). The drug droplets were then trapped in the secondary anchors adjacent to the primary anchors.^22^ Once most of secondary anchors were occupied, non-trapped droplets were flushed out of the chip. This protocol yielded one-to-one pairing of primary (spheroid containing) and secondary (drug solution) droplets. Subsequently, the interfaces of the adjacent trapped droplets were destabilized to fuse them into larger droplets (Figures 3C and 3D). Again, perfluorooctanol (PFO) in oil (FC40 + 20% PFO) was used as an emulsion destabilizer. Once droplet fusion was completed, oil without PFO (FC40) was flowed into the microfluidic chip to remove any remaining PFO, thus avoiding its interaction with cells.

To distinguish distinct drug concentrations for each spheroid, a barcode strategy was implemented using fluorescent dyes (Figure 3E). This was done by co-encapsulating etoposide with the Cascade blue dye (6 μM, fluorescent in the blue channel, Thermofisher) and cisplatin with the CF647 dye (1 μM fluorescent in the far red channel, Biotium). By mixing the dyes with the stock drug solutions, the concentration of the dye could be used as a measure of the drug concentration for the different dilutions. As the fluorescence intensity from these solutions would scale directly with dilutions of the stock solution, the fluorescence intensity measured on droplets of the drug stock was used for determining the drug concentration in each of the droplets. The calibration curves for the drug concentration and its fluorescence signal when used in our microfluidic platforms are shown in Figure S3. The calibration test demonstrated that the fluorescent signal scaled with the concentration of the dye (thus that of the drug) and that no interference with other fluorescent channels was found (no fluorescence cross talk). However the Cascade Blue dye did not provide a consistent calibration range on 3 decades of concentrations. As a result the etoposide range was split into two chips for each experiment, one with a low-concentration library and one with a high-concentration library.

### Single-drug toxicity on EwS spheroids

**Figure 4:**
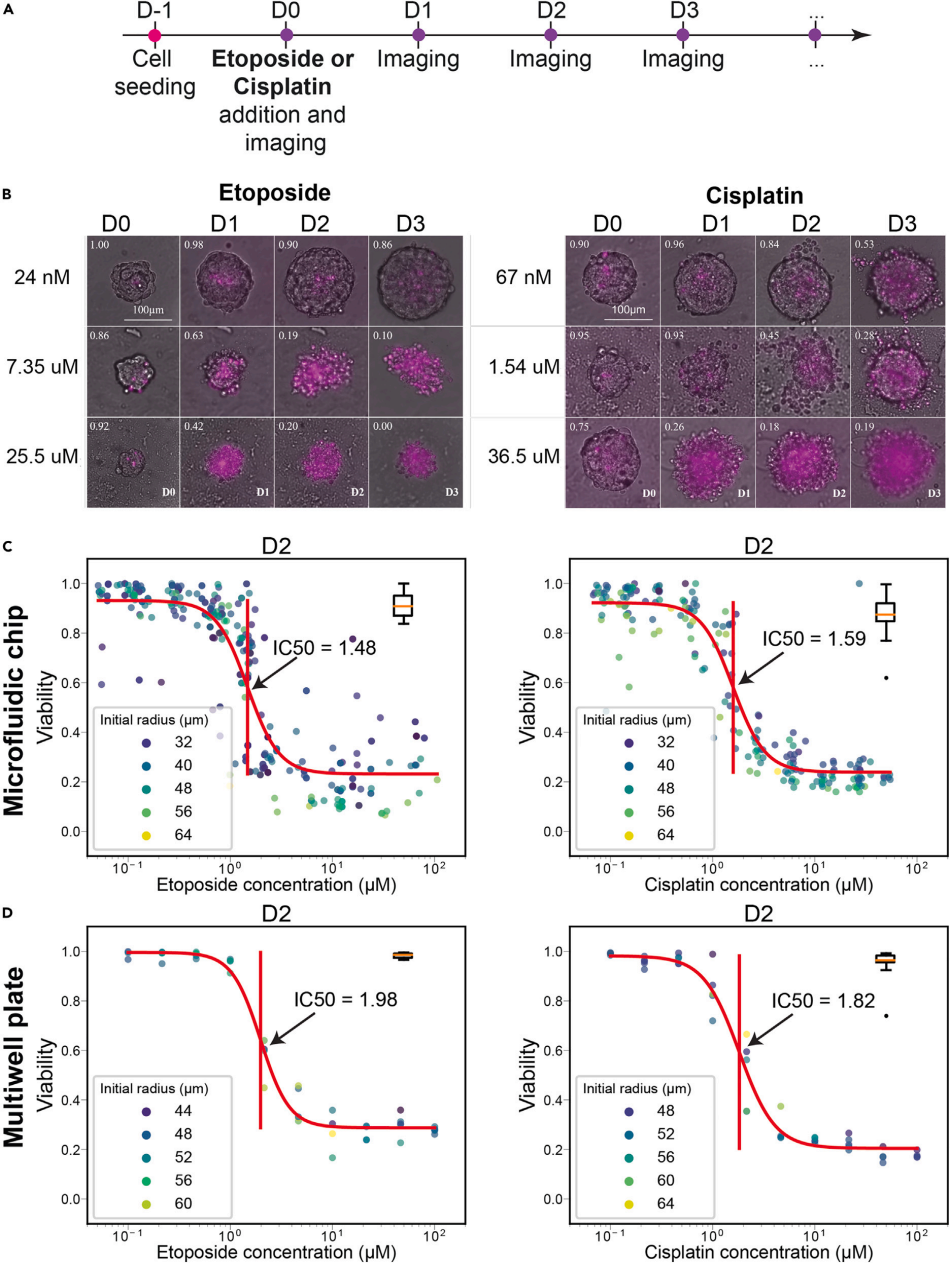
Effect of individual drugs on EwS spheroids (A) Timeline of the assay. Cells are seeded and 24 h later, when EwS spheroids are formed, they are exposed to a specific drug concentration and imaged every 24 h. (B) Representative images of spheroids over time (4 days), being exposed to different concentrations of either etoposide or cisplatin. Each image is a superposition of bright-field and PI fluorescent channel (shown in magenta), which indicates dead cells. (C) Viability quantification of EwS spheroids related to drug concentration using the microfluidic protocols. A sigmoidal fit is performed over these data to determine the IC50 value (indicated with a vertical bar in the middle of the sigmoid). The color of plotted dots indicate the measured spheroid radius in micrometers. On the right side of each plot, a boxplot represents the viability of the control group, subjected to either DMSO or NaCl. (D) Corresponding drug response curves obtained on spheroids in 96 well low-attachment plates.

The pipeline presented above was first used to measure the individual toxicity of either etoposide or cisplatin on EwS spheroids. The experimental timeline is shown in Figure 4A: On the first day of the experiment (D-1), approximately 200 A673 cells were introduced into the droplets in the culture device to form EwS spheroids. 24 h later (D0) secondary droplets representing 10 drug dilutions over three orders of magnitude of either etoposide or cisplatin were added to the first spheroid containing droplet using the barcoding strategy, resulting in final concentrations ranging from 40 nM to 200 μM. In parallel with the drug-containing droplets, control droplets were introduced in each chip (3% DMSO final concentration for the etoposide and 0.3% NaCl for the cisplatin) and labeled with the CF488A dye (green, Biotium, 0.3 μM in the final droplets). A first image of the complete microfluidic chip was obtained immediately after the drug addition to read the barcode on each of the spheroids. This allowed us to assign a drug concentration for every position within the chip. Imaging was then performed every 24 h on D1, D2 and D3, with the microchannels incubated in a cell culture incubator in the meantime. Sample images for each of the two drugs are shown in Figure 4B and show the increase of PI positive cells as well as the destruction of the spheroids for high drug concentrations.

This experimental protocol was then coupled with the image analysis pipeline to obtain the viability of each spheroid. Although individual spheroids showed some heterogeneity even for the same conditions, the pooled data allowed a precise determination of the IC50 value of the drugs for each of the culture days (See Figure S4 for complete datasets). These data were fitted with a sigmoidal function to determine the IC50 value, as shown in Figure 4C for D2. These experiments were repeated in parallel using spheroids cultured in standard 96-well low-attachment plates to benchmark the microfluidic results against the standard protocol. The results for D2 on spheroids cultured in 96-well plates are shown in Figure 4D and full results of these experiments are shown in Table 1. The IC50 values found in plates and in the microfluidic experiments were in very good agreement, as shown in Table 1 (see Table S1 for full results). The agreement between the two formats indicates that the microfluidic format does not introduce any strong bias on the measurements of the IC50 over the experimental periods studied here.

**Table 1:** IC50 values of cisplatin and etoposide against EwS spheroids measured on chip and on multiwell plates

IC50 values measured on chip versus multiwell plate				
Measurement timepoint	Etoposide		Cisplatin	
	Plate	Chip	Plate	Chip
D1	10.7 μM	9.84 μM	12.6 μM	14.66 μM
D2	1.98 μM	2.93 μM	1.82 μM	2.74 μM
D3	0.95 μM	0.95 μM	0.71 μM	1.06 μM
D4	N/A	0.73 μM	N/A	0.85 μM

IC50 values measured on multiwell plates (1 plate with 60 spheroids) and in microfluidic droplets (average value on several biological replicates: 4 independent experiments with multiple chips to screen the entire drug range, resulting in a total of 17 chips per drug, see Figure S4 for complete datasets).

### Combinatorial screening on EwS spheroids

Following the validation of the single-drug screen, combination of etoposide and cisplatin against EwS spheroids was evaluated with the same fluorescent barcoding strategy as the single drug experiments. The use of two fluorescent dyes allowed us to combine the two drugs on the same microfluidic device, while keeping track of the contents of each droplet on the chip. Three different configurations were used: (1) Simultaneous addition of cisplatin and etoposide (D1), (2) addition of cisplatin at D1 then etoposide at D2, (3) addition of etoposide at D1 and then cisplatin at D2. For all these different configurations, two steps of secondary droplet injection/fusion were performed because each secondary droplet came from a different library, generated in the aforementioned device.

The drug combination was investigated in the 30 nM to 30 μM and 10 nM to 10 μM concentration ranges for cisplatin and etoposide, respectively. Within these ranges, 6 drug concentrations were tested for etoposide, and 7 for the cisplatin. For a given combinatorial configuration, four to five chips were injected with the library of cisplatin. Then, two of these chips were injected with the etoposide low-concentration library and two to three chips were injected with the etoposide high-concentrated library. Some spheroids were subjected to a single drug, because some primary droplets fused with only one secondary droplet or with one droplet containing single drug and a second droplet containing a control solution (DMSO or NaCl). This enabled us to obtain IC50 values for both etoposide and cisplatin alone. Consequently the spheroids were exposed to 56 combinatorial conditions in a single run.

**Figure 5:**
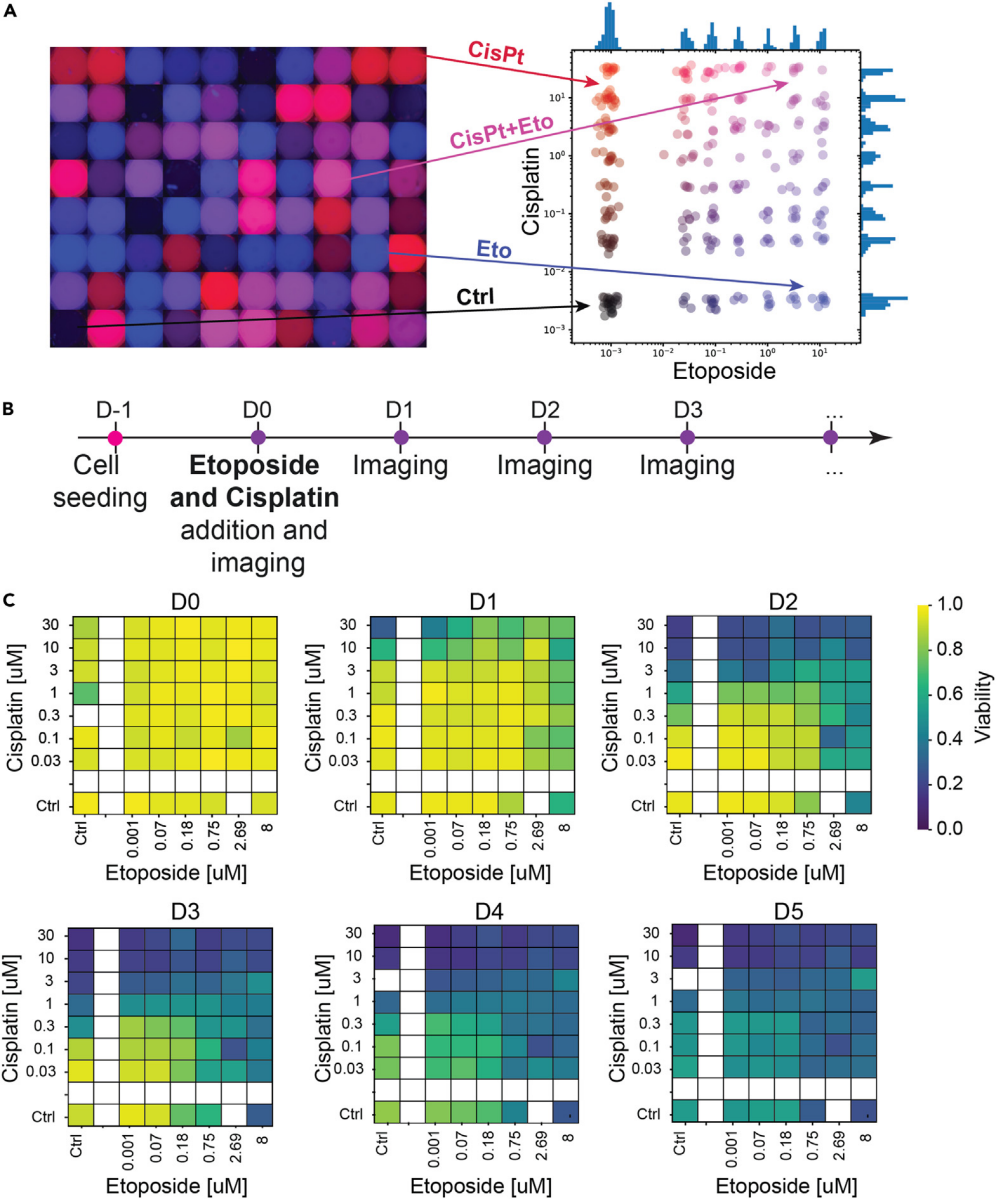
Simultaneously combined drugs on EwS spheroids (A) Two-color fluorescence image of each well after the addition of the secondary droplet allows a measurement of the drug concentrations in each well. Right: Representation of each condition on a 2D parameter space. Each dot represents one spheroid tested at a given combination. Controls with one drug or no drug are also included in this visualization. The experiment was performed using 4 chips, containing a total of 320 droplets. (B) Timeline of the assay. Cells are seeded and 24 h later, when EwS spheroids are formed, they are exposed to both drugs at specific concentrations and imaged every 24 h. (C)EwS spheroids viability heat maps over 6 days. The color of each square represents the averaged viability in the corresponding concentration range.

The concentration of each of the two drugs could be retrieved for each droplet by performing a two-color fluorescent readout, as illustrated in Figure 5A. The conditions can then be represented in a 2D parameter space, where etoposide concentration is represented in the xaxis (in blue), whereas cisplatin is represented in the yaxis (in red). Combinations of both drugs are presented in shades of violet. Controls are presented along either of the axes and the double-negative control is represented in black.

### Simultaneous assay

The effect of drugs applied simultaneously was tested on the viability of EwS spheroids, following the timeline shown in Figure 5B. First, EwS spheroids were formed in an array of droplets. This was followed 24 h later (D0) by the addition of droplets from the drug library to the spheroid-containing droplets. With the control droplets of each drug library, the spheroids received either a combination of both drugs, one drug and one control droplets or two control droplets. Subsequentwide field imaging was performed every 24 h in 5 channels (Brightfield, DAPI, FITC, TRITC and CY5) to determine the drug concentration, shape and viability of the EwS spheroids over time.

The results of the experiment are presented in Figure 5C, where each heatmap represents the mean viability per drug concentration in one single time point after drug addition. Controls are presented along the xaxis for etoposide and yaxis for cisplatin. From the panels, we detected a progressive increase in mortality over the days, which is more rapidly observed in samples challenged with the high drug concentrations (right and top side of the heatmaps). Spheroid mortality under cisplatin was found to occur faster than under etoposide, as can be seen when comparing the controls (x versus y axis).

### Sequential assay

**Figure 6:**
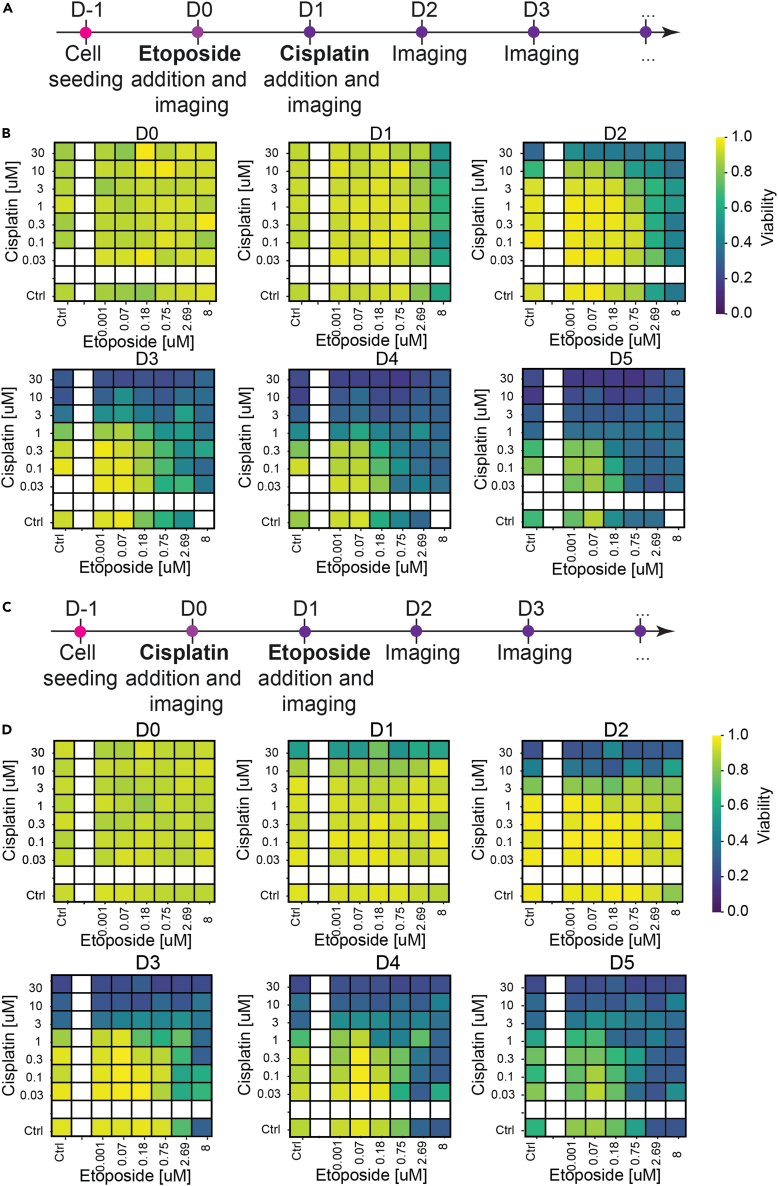
Sequentially combined drugs on EwS spheroids (A) Timeline of the cisplatin-etoposide sequential assay. Cells are seeded and 24 h later, when EwS spheroids are formed, they are exposed to etoposide and imaged. 24 h later, spheroids are exposed to cisplatin and imaged. Subsequently, they are imaged every 24 h. (B) EwS spheroids viability heatmap per day, according to and averaged in discreet concentration combinations. 4 chips were used, corresponding to 240 droplets. (C) Timeline of the etoposide-cisplatin sequential assay. (D) EwS spheroids viability heatmap per day, according to and averaged in discreet concentration combinations. 5 chips were used, corresponding to 320 droplets.

We next assessed the effect of applying the chemotherapies to EwS spheroids in a sequential manner, with a delay of one day between addition of the first and the second drug. The two experimental protocols with their corresponding heatmaps are shown in Figures 6A–6D.

The spheroid viability data display an asymmetric evolution, with the first drug starting to demonstrate an effect one day after administration, followed by the effect of the second drug on later days. When coupled with the different dynamics of action of the two drugs, this leads to different drug-response dynamics in the two protocols. As a result, the viability data from the two protocols are similar at late days (e.g. D5) but differ markedly at early days (e.g. D2).

### Synergy between drugs

Combination drug treatments aim, beyond the simple addition of the individual drugs, to identify synergies between the different drugs that provide therapeutic advantages over single treatments. Such synergies can be detected through large clinical studies.^35^ *In vitro*, different measures exist to identify the synergistic or antagonistic effects between two drugs, such as the effect addition, Bliss independence, or Loewe additivity, as described in detail in ref. ^36^. Here we choose to follow the protocol described in ref. ^37^, by obtaining the Loewe additivity measurements for the different conditions. This method has the advantage of being simple to implement and to provide a numerical answer for the combination of two drugs.

**Figure 7:**
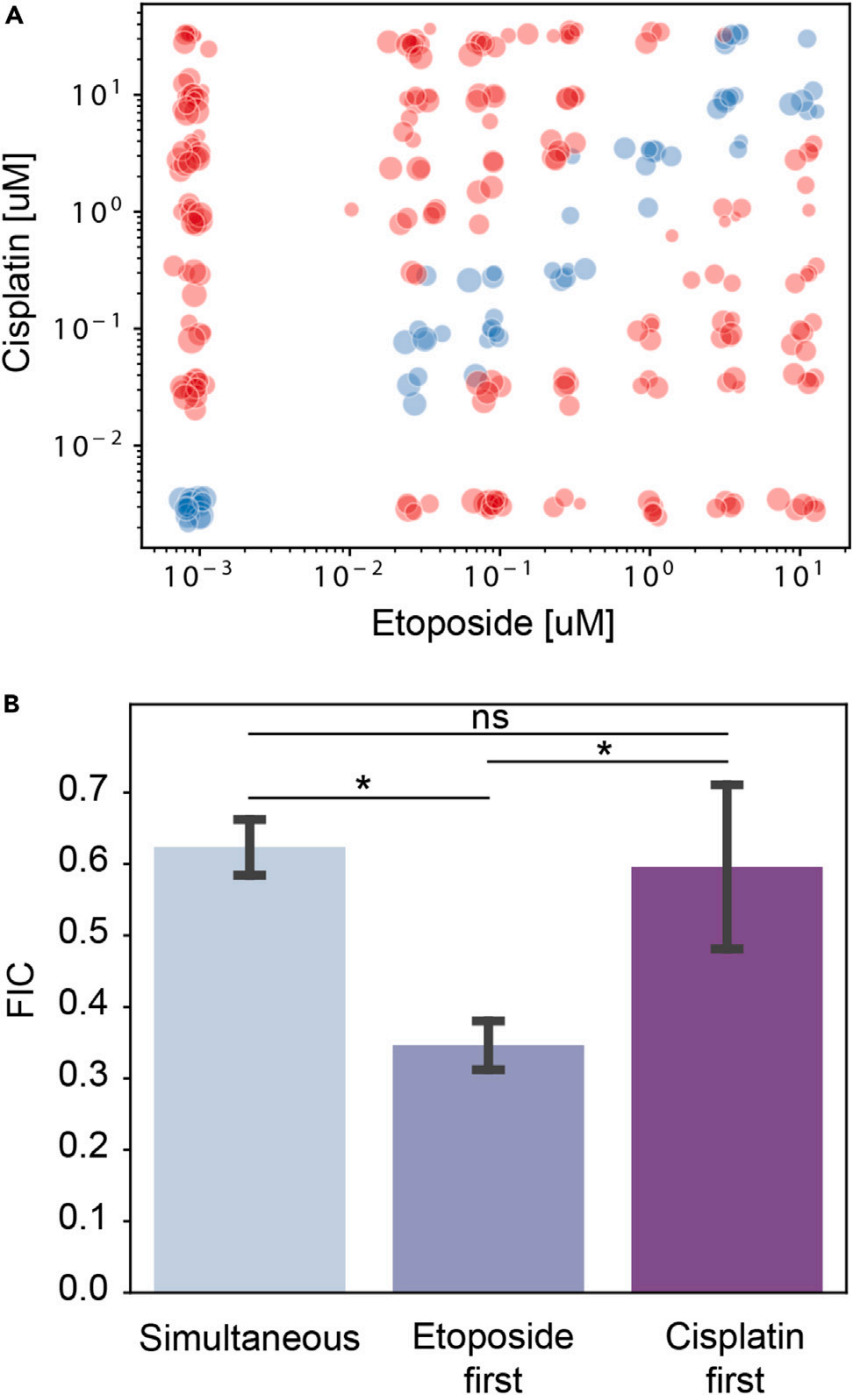
Detecting synergy between the drugs (A) The points along the diagonal of the two drug concentration are used to obtain a value of the IC50 of the drug combination. (B) The FIC index of the three conditions shows a synergy in all cases, with the etoposide condition displaying the strongest synergy. Data are represented as mean +/− standard deviation. The statistical analysis relies on a Wilcoxon-Mann-Whitney test. n.s.: non-significant; ∗: p<0.05.

The Loewe method compares the IC50 for the combined experiments with its value for a single drug by focusing on the diagonal in the 2D parameter space, as shown by the blue dots in Figure 7A. The viability at each of the concentrations along the diagonal is used to obtain the IC50 value for the combination of drugs. The value of the IC50 is then divided by the mean value of the IC50 for the two drugs alone to obtain the Fractional Inhibitory Concentration (FIC). Values of the FIC>1 indicate that the drugs are antagonistic, whereas values of FIC<1 indicate synergy between the drugs.^37^

In the current study, three FIC numbers can be compared together, corresponding to the three combination experiments: simultaneous (S), etoposide first (E), and cisplatin first (C). The values of the FIC for these three conditions are shown in Figure 7B. All three values are indeed smaller than one, indicating synergistic interactions found when drugs are applied simultaneously or sequentially. Of interest, synergistic interactions were found more effective when etoposide was applied first.

## Discussion and outlook

In the present study, we demonstrate a protocol to screen combination therapy on spheroids within an array of droplets. The microfluidic droplet array provides a format that is well suited for spheroid culture and observation, particularly to follow the evolution of each spheroid as a function of time.^14^^,^^20^^,^^32^ Then the ability to merge successive droplets with the initial spheroid-containing drops enables a large versatility of experimental protocols with only a minor increase in the protocol complexity.^22^ This is demonstrated by applying simultaneous or sequential drug combination screens while using the same experimental and analytical pipeline.

These results echo combination results obtained using segmented flow assays to perform coupled screens, by taking advantage the strong flexibility in determining the droplet contents both for micro-organisms^38^ and for cancer cells.^39^ Indeed previous methods have been published for screening combined conditions on cancer cells, either using water-in-oil to encapsulate the different conditions^39^^,^^40^ or using parallel channels with flow-control.^41^^,^^42^ Although each of the above approaches has its specific advantages, the method presented here is unique in that the microfluidic device is disconnected from flow control for most of the experiment. As such a single flow control unit can be used to inject droplets in a multitude of microfluidic devices in parallel, which is shown here by running several chips in parallel for most conditions. The devices are then disconnected and stored in a cell culture incubator and imaged on a regular microscope. This “no-flow” condition greatly simplifies the operation of the microfluidics and allows for scale-up to high-throughput platforms. Although the droplet volume in the current platform is limiting for large spheroids or experimental protocols beyond a few days, larger droplet volumes can be implemented by using larger microfluidic anchors. The resulting increase in volume will provide a larger reservoir that automatically support longer term cell culture.^14^

The results shown here have direct applications for both clinical and fundamental research. First regarding clinical applications, the platform that we demonstrate here will allow us to take patient-derived samples and test them against different drug treatments, in the context of personalized cancer medicine or companion testing. Working with 3D cultures, namely as spheroids or organoids, will provide an opportunity to improve the relevance of the *in vitro* model for recapitulating the structure of the initial tumor.

In the case of fundamental scientific studies, the format of anchored microfluidic droplets has already been shown to be well-adapted for performing single-cell measurements that are resolved in both space and time.^20^^,^^22^^,^^23^ This ability to probe the response of individual cells within the spheroids to the drugs provides a method to identify the fundamental mechanisms the lead to the synergy of antagonism between drugs, for example through the use of live-cell measurements. Indeed, the approach can be combined with cell-therapy modeling^23^ to allow the screening of combined cellular and chemotherapy strategies.

### Limitations of the study

This study uses spheroids of a cancer cell line to demonstrate the microfluidic platform that allows the screening of drug combinations. As such the implications of the measured biological response beyond this model system must be treated with care. Moreover, the small volume associated with the droplet format may impact the viability measurements after several days in culture. We have validated that the cells remain viable in the absence of drug treatment and that the IC50 after two days of culture in droplets matches the standard multiwell plates. Nevertheless dying cells may secrete by-products that may influence their neighbors and this effect may be more pronounced in the small droplets. The implications of this confinement to understanding drug response *in vivo* would be interesting to investigate.

## STAR★Methods

### Key resources table

**Table:** 

REAGENT or RESOURCE	SOURCE	IDENTIFIER
**Chemicals, peptides, and recombinant proteins**		

Etoposide	Merck – Sigma-Aldrich	E1383
Cisplatin	Merck – Sigma-Aldrich	232120
CF647	Biotium - Sigma-Aldrich	SCJ4600048
CF488A	Biotium- Fisher Scientific	50-196-4622
Cascade Blue	Molecular Probes, Fisher Scientific	11550166
FC40 oil	3M -Inventec	99687220
RAN Fluorosurfactant	Ran biotechnologies	008-FluoroSurfactant

**Deposited data**		

Raw data and analysis code	This paper	https://github.com/BaroudLab/screening-spheoroids-analysis

**Experimental models: Cell lines**		

A673	ATCC	CRL-159

**Software and algorithms**		

Python version 3.9	Python Software Foundation	https://www.python.org
ImageJ version 1.53q	ImageJ	https://imagej.nih.gov/ij/

### Resource availability

#### Lead contact

Further information and requests for resources and reagents should be directed to and will be fulfilled by the lead contacts, Prof. Charles N. Baroud (charles.baroud@pasteur.fr).

#### Materials availability

This study did not generate new unique reagents.

### Experimental model and subject details

#### Cells and reagents

The A673-GFP cell line was derived from the A-673 cell line (ATCCCRL-1598) by transduction with a plasmid vector, pCDH1-eGFP encoding enhanced green fluorescent protein and kindly provided by Dr. K. Laud-Duval (U830 - InstitutCurie, Paris). The cells were cultured in a CO ${}_{2}$ incubator (5% of CO ${}_{2}$, 37°C, C150, C150), on T-25, 25 cm^2^ cell-culture flask (Corning) in 5 mL of high glucose Dulbecco’s modified Eagle’s medium (DMEM + GlutaMAX, ThermoFisher), supplemented using 10% of Fetal Bovin Serum (FBS, ThermoFisher) and 1% of penicillin-streptomycin (ThermoFisher). Upon 80% of confluency, cells were then subcultured - twice a week - according to the following protocol: (i) In the flask, culture medium was rinsed using Phosphate Buffered Saline solution (PBS, ThermoFisher) (ii) cells were detached using 500 μL of trypLE Express (iii) Finally, once detached, approximately 50 000 cells were seeded in 5 mL of supplemented medium (approximately 1:20 ratio) within a 25 cm^2^ cell-culture flask. In this study, we used two different drugs: cisplatin and etoposide. Pure cisplatin powder was dissolved at 10 mM in a 0.9% NaCl aqueous solution, and pure Etoposide was diluted in DMSO at 3.3 mM.

### Method details

#### Microfabrication

Molds were mainly fabricated using standard dry film soft lithography. Up to five layers of dry film photoresist, consisting of 50 and 33 μm Eternal Laminar (respectively E8020 and E8013, Eternal Materials, Taiwan) and 15 μm AlphoNIT215 (Nichigo-Morton) negative films, were successively laminated using an office laminator (PEAK pro PS320) at a temperature of 100°C until the desired channel height, from 50 to 200 μm depending on the different cases, was reached. After each laminating step, the photoresist film was exposed to UV (LightningCure, Hamamatsu) through a photomask of the junction, channels, trapping chamber boundaries or anchors. The masters were revealed after washing in a 1% (w/w) K2CO3 solution (Sigma-Aldrich). The top of the chip consisted of the flow-focusing device and chambers and the anchors were located at the bottom of these chips. The anchors mold was designed with RhinoCAM software (MecSoft Corporation) and was fabricated by micro-milling a brass plate (CNCMini-Mill/GX, Minitech Machinery). That was also the case for the droplet library producing chips. The topography of the molds and masters were measured using an optical profilometer (VeecoWycoNT1100, Veeco). For the fabrication of the top of the chip, poly(dimethylsiloxane) (PDMS, SYLGARD 184, Dow Corning, 1 g of curing agent for 10 g of bulk material) was poured over the master and cured for 2h at 70°C. The metallic mold was first covered with PDMS. Then, a glass slide was immersed into uncured PDMS, above the anchors. The mold was finally heated on a hot plate at 180°C for 15 minutes before extraction of the glass slides covered by a thin layer of PDMS with the anchor pattern. In all cases, the top and the bottom of chip were sealed after plasma treatment (Harrick). Eventually, the chips were filled 3 times with Novec Surface Modifer (3M), a fluoropolymer coating agent, for 30min at 110°C on a hot plate.

#### Microfluidic protocol

The loading of the first droplet was made using a 2% solution of surfactant diluted in FC40 oil. All air bubbles were discarded. A673 cells were detached from the culture flasks with a 2-3 minutes incubation in TrypLE^TM^ Express enzyme (ThermoFischer), that was then inactivated by addition of warm medium. The cell concentration was determined using a haemocytometer and adjusted to $4.10^{5}$ cells/mL. For enabling the quantification of the cell viability, propidium iodide (ThermoFisher) was introduced at a concentration of 1μM. One glass syringe was loaded with this solution and droplets were produced using neMESYS syringe pumps (Cetoni) as previously described. After the loading, the chips were kept immersed in PBS in the CO2 incubator.

#### Library fabrication and barcoding

Libraries were produced in a dedicated microfluidic chip, based on gradient of confinement (2). For a given solution, a plug of 25 μL of aqueous phase was split in droplets presenting a volume of 20 nL. For avoiding cross contamination between two successive plugs, the plugs were separated by a succession of 2 μL of oil, 2 μL of air, and 2 μL of oil. HFE 7500 (3M) constituted the oil phase, and the emulsion was stabilized thanks to 3% of the fluorosurfactant (RAN Biotechnologies).

The initial solution of cisplatine was concentrated at 3.3 mM in 0.9% of NaCl in water. The corresponding control was a solution of the fluorescent dye CF488A (Biotium) at 0.3 μM diluted in 0.9% NaCl in water solution. The drug solution was labeled using the fluorescent dye CF647 (Biotium, 1 μM maximum final concentration). Etoposide stock solution of 10 mM was prepared in DMSO. Etoposide library contains a control solution of the fluorescent dye CF488A (Biotium) diluted at 6 μM maximum final concentration in 3% DMSO. Etoposide library contains a control solution of the fluorescent dye CF488A (Biotium) diluted at 6 μM maximum final concentration in 33% DMSO solution, corresponding to the highest concentration of DMSO achieved in our study with the most concentrated Etoposide solution. After merging of the first and secondary droplets, the control solution represents a final volumic concentration of DMSO slightly smaller than 1%. A plug of 12.5 μL (half the volume of a dilution plug) of the control solution was added to each library.

#### Image acquisition

Images without cells were acquired using a binocular microscope (Nikon SMZ18) with a digital single-lens reflex camera (D7000, Nikon). The fluorescence images of the spheroids were taken with an inverted microscope (Eclispe Ti, Nikon), equipped of a motorized stage, an illumination system (Spectra-X, Lumencor) with a CMOS camera (ORCA Flash 4.0, Hamamatsu). The images were acquired with a 10x Plan-Apo objective (NA = 0.45).

### Quantification and statistical analysis

#### Viability analysis

The fluorescent images were acquired using a classical epi-fluorescence microscope. Therefore, in fluorescence, each pixel integrates some signal from above and below the focus plane. In order to take this into account for the propidium iodide (PI) signal, we designed a viability calculation that combines an objective thresholding and signal integration. First, a mask of the entire spheroid is obtained by combining 2 masks: one by applying an Otsu threshold (using a native Matlab function) on the green fluorescent image, and one obtained by thresholding the PI image. This way, the overall mask represents the entire spheroid, with live and dead cells. The PI threshold is set as follows to obtain the PI mask:

(Equation 1) $$Threshold\;PI=median\left(PI_{m}\right)+2\sigma \left(PI_{m}\right)$$

where $PI_{m}$ is the orange fluorescence value over the complete field of view and σ represents the standard deviation.

Second, the PI fluorescent intensity is integrated over this PI mask. The mortality ratio is obtained by dividing this integral value by the theoretical integral PI value of a spheroid of identical area which would be 100% dead. This theoretical integral is calculated by multiplying the *Area* of the spheroid (calculated on the overall mask) by a normalization factor *K* that can be seen as the integral PI value that would be obtained on a single column of pixels in the completely dead spheroid. *K* is estimated by adding 2 standard deviation to the mean PI signal of the pixels above the fluorescent threshold calculated above. Therefore, the viability is calculated as follows:

(Equation 2) $$Viability=1-\frac{\int PI}{K\times Area}$$

This method is graphically explained on Figure S5. This viability calculation does not rely on any user input and has proven high consistency with the images of this study. Using this method, size and viability of the spheroids was scored every 24 hours over the course of experiments. A representative image-series of a spheroid is shown in Figure 2D.

#### Statistical analysis

The statistical analysis of Figure 7B relies on a Wilcoxon-Mann-Whitney test. n.s.: non-significant; ∗: p<0.05.

## Data Availability

• All data reported in this paper will be shared by the lead contact upon request. • All original code has been deposited on GitHub and is publicly available as of the date of publication by using the following link: https://github.com/BaroudLab/screening-spheoroids-analysis. • Any additional information required to reanalyze the data reported in this paper is available from the lead contact, Charles Baroud (charles.baroud@pasteur.fr) upon request.
