# Supplementary material for: Combinatorial drug screening on 3D Ewing sarcoma spheroids using droplet-based microfluidics
Source: iScience. 2023 Apr 12;26(5):106651. doi: 10.1016/j.isci.2023.106651 (PMC10165258; doi:10.1016/j.isci.2023.106651)
Supplement: Document S1. Figures S1–S5 and Table S1 [file mmc1.pdf]

iScience, Volume *26*

Supplemental information

Combinatorial drug screening on 3D Ewing sarcoma spheroids using droplet-based microfluidics

Romain Fevre, Gaëtan Mary, Nadia Vertti-Quintero, Aude Durand, Raphaël F.-X. Tomasi, Elaine Del Nery, and Charles N. Baroud

SUPPLEMENTAL FIGURES

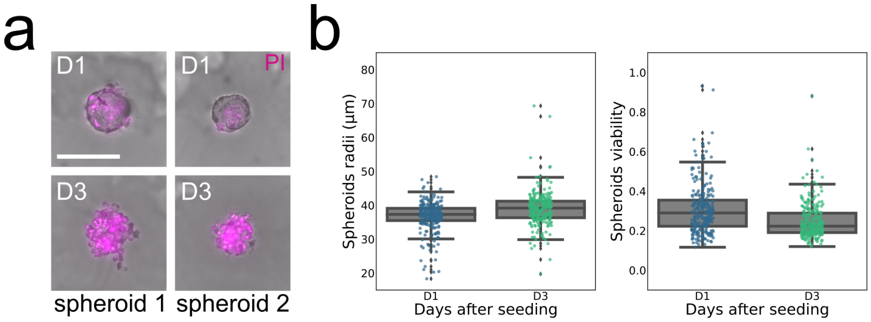

FIG. S1 **A673 spheroids grown in 45 nL droplets.** Related to Figure 2. **(a)** Representative images of two spheroids grown in 45nL droplets, 24h and 72h after the seeding, stained with PI. scale bar: 100µm. **(b)** Spheroid radii over time (in days) when cultured in 45 nL droplets, as well as their viability over time. n=251 droplets

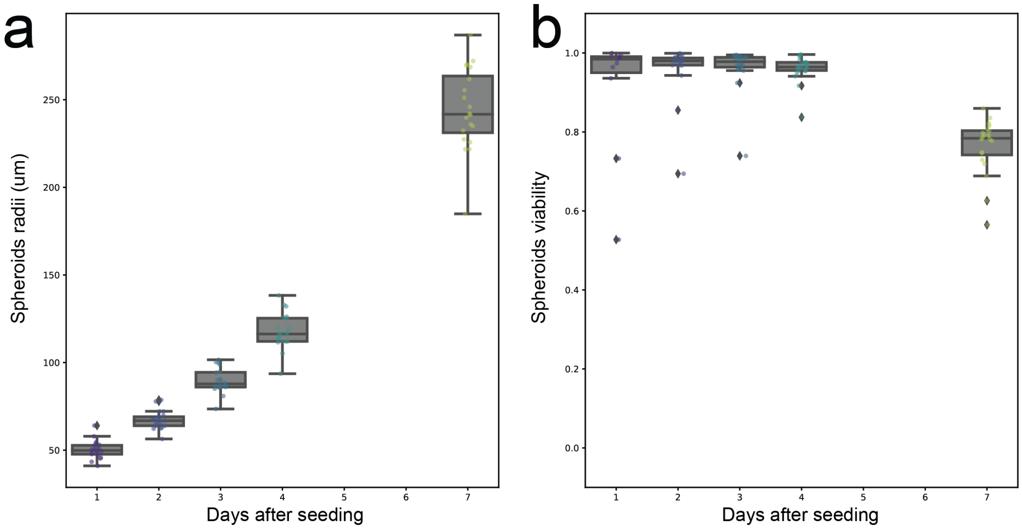

FIG. S2 **Growth and viability measurements of spheroids in multiwell plates.** Related to Figure 2. **(a)** Radii of EwS spheroids measured every 24 hours on a multiwell plate. **(b)** Viability of EwS spheroids measured every 24 hours on a multiwell plate.

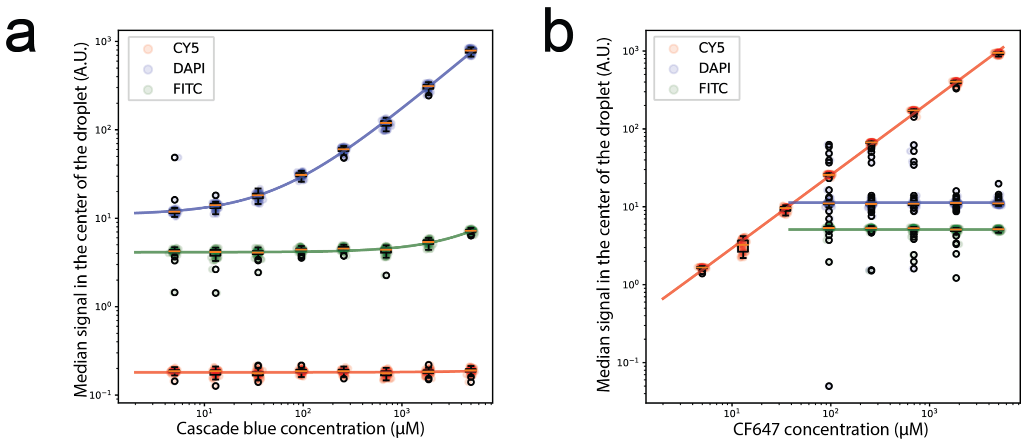

FIG. S3 **Calibration curves for barcoding.** Related to Figure 3. Calibration of fluorescent solutions used for barcoding: **(a)** Cascade blue and **(b)** CF647. Dots in plots represent the levels of intensity, normalized by exposure time: in blue DAPI channel, green FITC and red for CY5. The colored lines represent linear fits of the data. The fluorophores cited in the legend refer to the filter sets rather than the actual fluorescent molecules.

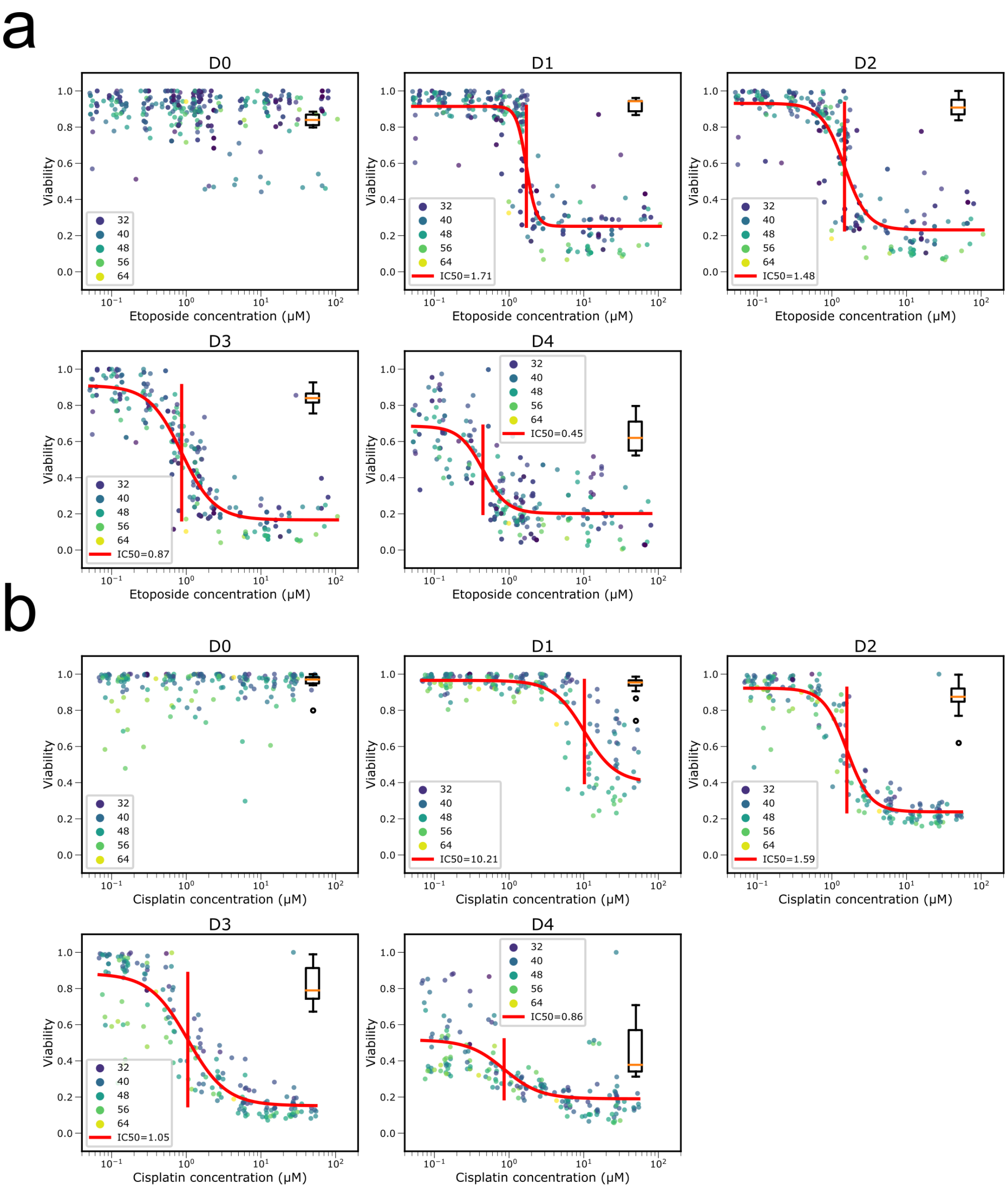

FIG. S4 **Viability of EwS spheroids per drug concentration supplied.** Related to Figure 4. Viability of spheroids depending on drug concentration supplied along four days after drug supply for IC50 value determination per day: **(a)** etoposide and **(b)** cisplatin. On the right side of each plot, a boxplot represents the control group. The colorsmap of the markers corresponds to the initial radii of the spheroids (μm)

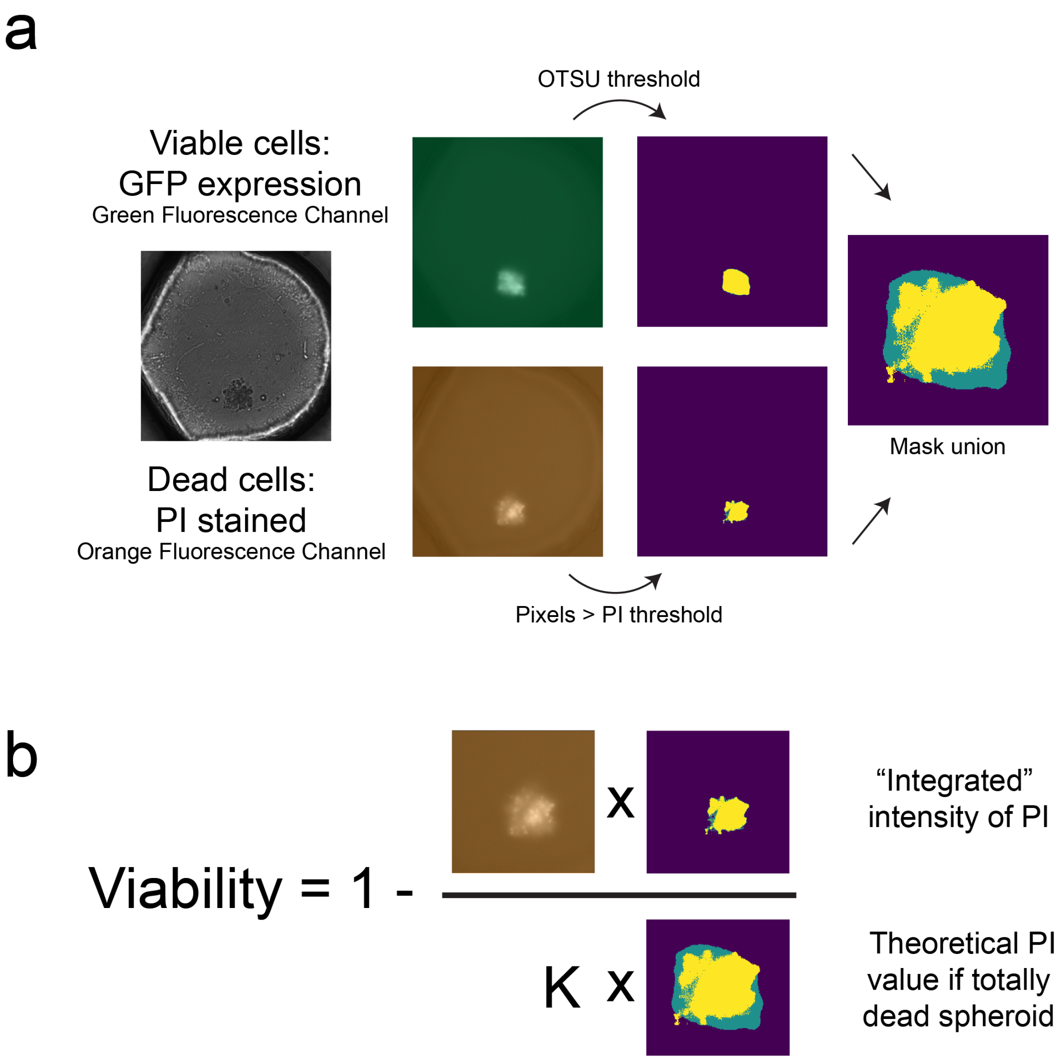

FIG. S5 **Pipeline for viability scoring through image analysis.** Related to Star methods Eq. 2. **(a)** Images are segmented over the fluorescence channels according to their fluorescence values: The total cells are found with an OTSU threshold (native Matlab function) in the green fluorescence channel while the dead cells are found with the pixels whose value is larger than the PI threshold value. **(b)** The viability of the spheroid is calculated as follows: 1 - (The integrated PI value: the actual measured area with PI fluorescence from the orange fluorescence channel)/(K: The theoretical value of a complete dead column of cell times the complete area of the spheroid.)

SUPPLEMENTAL TABLE

IC50 values measured on chip vs multiwell plate										
Measurement timepoint	Etoposide					Cisplatin				
	Plate	Chip R1	Chip R2	Chip R3	Chip R4	Plate	Chip R1	Chip R2	Chip R3	Chip R4
D1	10.7 µM	1.71 µM	8.11 µM	13.18 µM	16.31 µM	12.6 µM	10.21 µM	18.45 µM	11.17 µM	18.76 µM
D2	1.98 µM	1.48 µM	3.14 µM	2.07 µM	4.93 µM	1.82 µM	1.59 µM	4.22 µM	1.38 µM	3.78 µM
D3	0.95 µM	0.87 µM	1.48 µM	0.49 µM	0.97 µM	0.71 µM	1.05 µM	1.27 µM	0.51 µM	1.42 µM
D4	n/a	0.45 µM	n/a	0.26 µM	0.61 µM	n/a	0.86 µM	n/a	0.35 µM	0.9 µM

TABLE S1 **IC50 values of etoposide and cisplatin against EwS spheroids measured on chip and on multiwell plates.** Related to Table 1. IC50 values measured on multiwell plates and in microfluidic droplets. Chips R1-R4 constitute biological replicates.
